# Fracture risk after intralesional curettage of atypical cartilaginous tumors

**DOI:** 10.1186/s13018-023-04215-4

**Published:** 2023-11-09

**Authors:** Gitte G. J. Krebbekx, Felix J. Fris, G. R. Schaap, J. A. M. Bramer, F. G. M. Verspoor, Stein J. Janssen

**Affiliations:** 1 Department of Orthopaedic Surgery and Sports Medicine, Amsterdam UMC, University of Amsterdam, Meibergdreef 9, Amsterdam, The Netherlands; 2 Amsterdam Movement Sciences, Musculoskeletal Health, Amsterdam, The Netherlands

**Keywords:** Orthopedic oncology, ACT, Fracture risk

## Abstract

**Introduction:**

The need for curettage of atypical cartilaginous tumors (ACT) is under debate. Curretage results in defects that weaken the bone potentially leading to fractures. The purpose of this study was to retrospectively determine postoperative fracture risk after curettage of chondroid tumors, including patient-specific characteristics that could influence fracture risk.

**Methods:**

A total of 297 adult patients who underwent curettage of an ACT followed by phenolisation and augmentation were retrospectively evaluated. Explanatory variables were, sex, age, tumor size, location, augmentation type, and plate fixation. The presence of a postoperative fracture was radiologically diagnosed. Included patients had at least 90 days of follow-up.

**Results:**

A total of 183 females (62%) were included and 114 males (38%), with an overall median follow-up of 3.2 years (IQR 1.6–5.2). Mean diameter of the lesions was 4.5 (SD 2.8) cm. Patients received augmentation with allograft bone (*n* = 259, 87%), PMMA (*n* = 11, 3.7%), or did not receive augmentation (*n* = 27, 9.1%). Overall fracture risk was 6%. Male sex (*p* = 0.021) and lesion size larger than 3.8 cm (*p* < 0.010) were risk factors for postoperative fracture.

**Interpretation:**

Curettage of ACT results in an overall fracture risk of 6%, which is increased for males with larger lesions.

**Supplementary Information:**

The online version contains supplementary material available at 10.1186/s13018-023-04215-4.

## Introduction

Atypical cartilaginous tumors (ACT) are benign lesions, most commonly occurring centrally in the medullary cavity of long bones. They are often found incidentally at the diagnostic pathway of musculoskeletal complaints [1]. ACT was previously classified as chondrosarcoma grade 1. The World Health Organization (WHO) changed the nomenclature in 2013 because of its benign clinical behavior and corresponding good prognosis (5-year survival rate of 83 to 99%) [2–8].

Treatment of an ACT has shifted in the past decennia from wide resection to intralesional curettage, and nowadays some institutions advocate active surveillance [9, 10]. Studies demonstrated that curettage in combination with adjuvants is as safe as wide resection in terms of oncological outcome [11, 12]. Intralesional curettage is therefore considered as treatment option for ACTs [13–17]. However, the need for curettage of an ACT is under debate, as it seems safe to observe these lesions with regular MRI; this safely differentiates ACT from malignant chondrosarcoma [9, 18–20].

Nevertheless, most centers still perform curettage of ACTs, to preclude malignant transformation to chondrosarcoma. However, curettage comes with inherent risk of surgical complications, perhaps without proven—oncological—benefit [12, 21]. Curettage is performed through a cortical window with a curette or high-speed burr followed by an adjuvant (e.g., phenolisation, cryosurgery). The remaining cavity is often filled with either cancellous bone chips, femoral head allograft, PMMA cement, or autograft to stimulate bone healing and reduce fracture risk. Prophylactic plate-screw osteosynthesis is used to bridge the weakened bone segment and allows healing of the defect [22]. A lower fracture risk was seen for patients that also undergo prophylactic plating compared to those who did not undergo prophylactic plating (14% vs. 5%)[12]. However, the disadvantages of plating are the potential need for plate removal, longer surgery time, and an increased infection risk [21, 22].

The overall fracture risks after curettage varies between studies. The current knowledge of the benign behavior of ACT in long bones and the negative side effects of curettage, such as fractures, should discourage curettage and motivate active surveillance [9, 12, 21]. For this reason, this study re-evaluated fracture risk after the curettage of atypical cartilaginous tumors. Second, the likelihood of fractures based on patient-specific characteristics is observed, to help identify patients at risk of a fracture.

## Patients and methods

### Study design

This retrospective cohort study includes all patients (18 years and older) with an ACT referred to our tertiary care orthopedic oncology referral center, treated between January 2008 and May 2019.

**Fig. 1:**
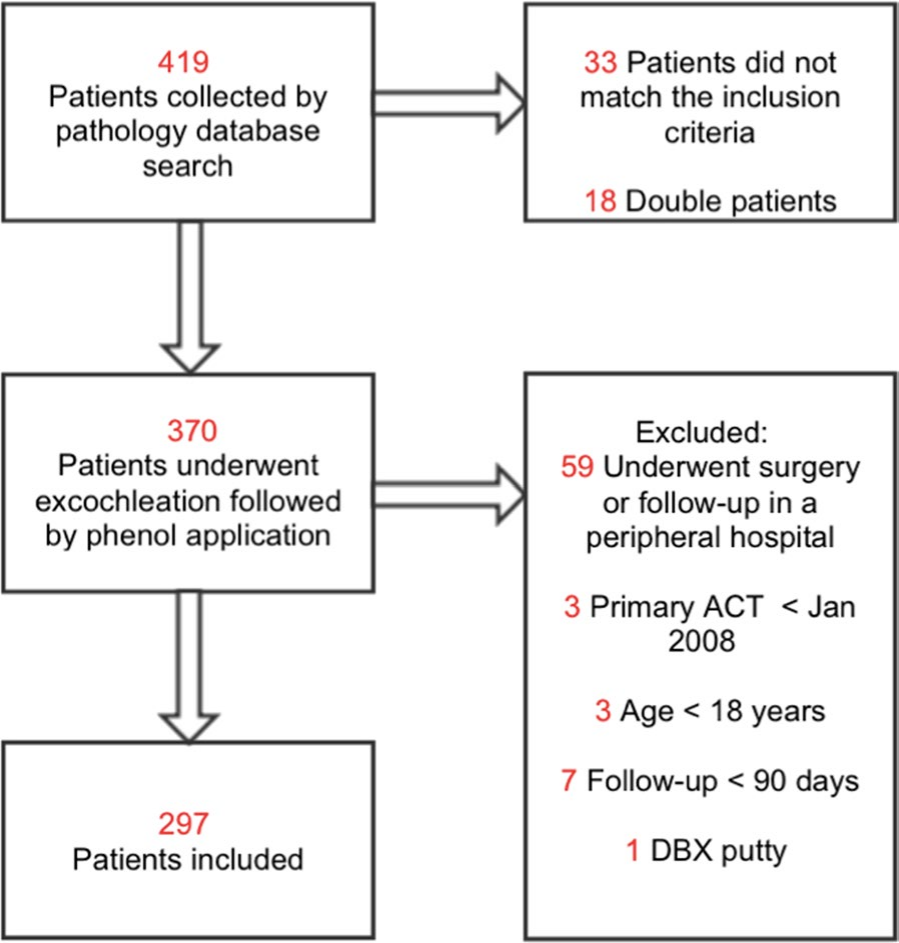
Flowchart, in-, and exclusion criteria

The pathology database was searched for keywords including ‘ACT’, ‘atypical cartilaginous tumor’, ‘chondrosarcoma grade 1’, ‘CS + 1’, ‘atypical cartilage + CS’, and ‘atypical chondroid + chondrosarcoma’. All medical records of identified patients were screened for eligibility. Exclusion criteria were follow-up less than 90 days (*n* = 16), or if augmentation other than allograft or PMMA (polymethyl methacrylate) was used (*n* = 3). If patients had multiple lesions on different sides, or a recurrence of the same lesion, only the first surgery was included per patient (*n* = 4), to not violate the statistical assumption of independence (Fig. 1).

The preoperative diagnosis of an ACT was based on clinical history, radiographs, and MRI, assessed at a multidisciplinary meeting. The meeting included at least a musculoskeletal radiologist, musculoskeletal oncology pathologist, radiation oncologist, medical oncologist, and oncologic orthopedic surgeon (all with extensive experience in bone tumors). Until 2019, surgical curettage was consistently performed in a standard manner using either a curette or a high-speed burr, based on the orthopedic surgeon’s preference. Whether or not augmentation in terms of defect filling (e.g., allograft, bone chips, PMMA cement) and/or plate fixation was used, was also at the discretion of the treating oncologic orthopedic surgeon. Tissue was sent for histological analysis, and all diagnostics (clinical history, imagines, pathology) were reviewed at the multidisciplinary meeting as mentioned above, to confirm the definitive diagnosis.

The standard postoperative follow-up schedule was at 6 weeks, 3 months, 6 months, 1 year, 2 years, and 5 years, after surgery. The median follow-up was 3.2 (IQR 1.6–5.2) years. Follow-up at one year was 86% (42/297 patients were lost to follow-up).

### Outcome measures and explanatory variables

The primary outcome measure was a postoperative fracture at the surgery site, which was clinically and radiologically confirmed and occurred within 1 year after surgery.

Explanatory variables included age, sex, preoperative fracture, plate fixation technique, location of the ACT, weight-bearing status postoperatively, type of augmentation, and length of ACT (intramedullary length) as described by the radiologist.

Location of the ACT was categorized into [1] affected bone (e.g., femur) and [2] part of the bone affected (i.e., proximal metaphyseal, diaphyseal, distal metaphyseal). Weight-bearing restrictions were categorized into full weight bearing, permissive weight bearing, 50% weight bearing, toe touch (10%) weight bearing, and non-weight bearing. All variations of restrictions were generally applied for 4–6 weeks, after which an X-ray was performed to inform further weight-bearing restrictions. Type of augmentation was categorized into allograft (including both cancellous bone chips and fresh cadaveric femoral head allograft) and PMMA.

### Statistical analysis

We used frequency with percentages to describe categorical variables and mean with standard deviation to describe continuous variables (as histograms suggest a normal distribution of continuous variables). Bivariate log-rank analyses were used to assess differences in fracture risk based on other explanatory variables. For statistical purposes, we used a median split to divide the cohort based on age (above and below 51 years) and tumor size (above and below 3.8 cm). A two-tailed *p* value below 0.05 was considered statistically significant. All analyses were performed using Stata® 16.0 (Stata Corp LP, College Station, TX, USA).

### Ethics, funding, and conflicts of Interests

This study protocol (reference number W21_572 # 22.014) was assessed by our institutional review board and was carried out following the applicable rules concerning the review of the research ethics committee and the Helsinki Declaration. No funding or benefits were received by any of the authors. None of the authors had a potential conflict of interest.

## Results

This study includes 297 patients, of which 62% (183/297) were women. The mean age was 51 (SD 12) years. The distal femur (40%) and proximal humerus (31%) were mostly affected (Additional file 1: Table S1). Tumor sizes ranged from 0.7 to 25 cm with a mean size of 4.5 cm (SD 2.8 cm). Nine patients (3%) had a preoperative fracture. Intraoperative prophylactic plate fixation was used in 110 (37%) patients. Allograft augmentation was used in 259 (87%) patients, 27 patients (9.1%) had no augmentation, and 11 (3.7%) had PMMA cement augmentation. Weight bearing was documented in 284 (96%) patients; most patients (*n* = 108, 36%) were non-weight-bearing directly postoperative. (Additional file 2: TableS2).

**Fig. 2:**
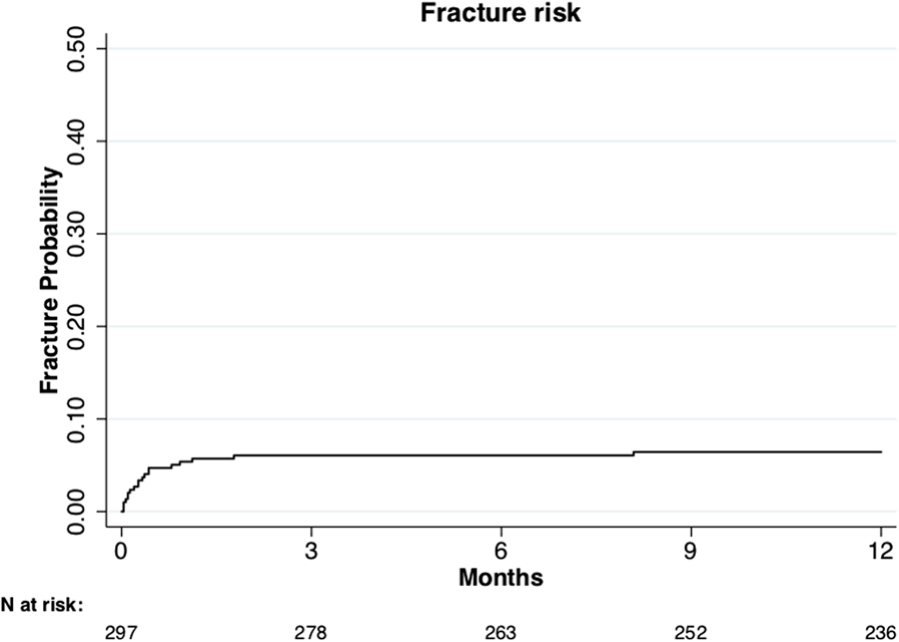
One-year fracture risk, overall

The overall fracture risk at 3 months and 1 year was 6% (probability: 0.06; 95% CI 0.04–0.10) (Fig. 2). The majority (95%; 18/19) of the fractures occurred within 2 months (Additional file 3: Table S3).

**Fig. 3:**
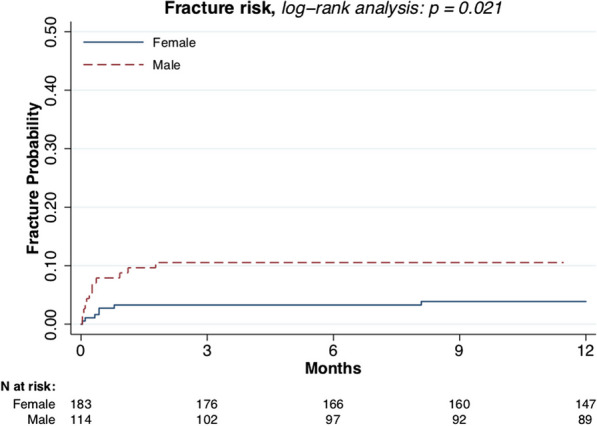
One-year fracture risk, sex

**Fig. 4:**
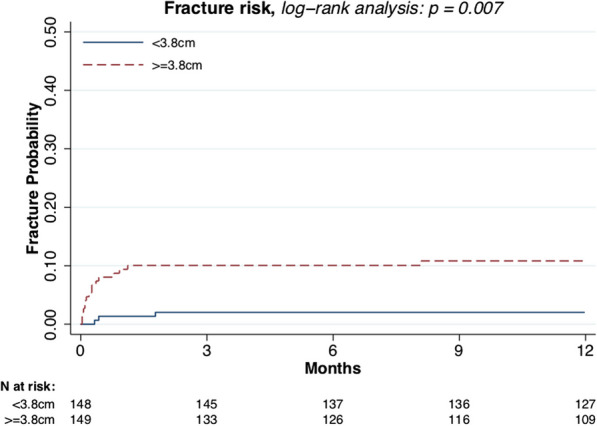
One-year fracture risk, size

**Fig. 5:**
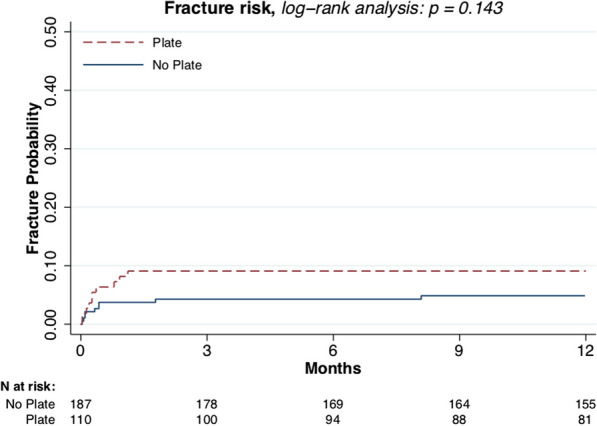
One-year fracture risk, plate fixation

**Fig. 6:**
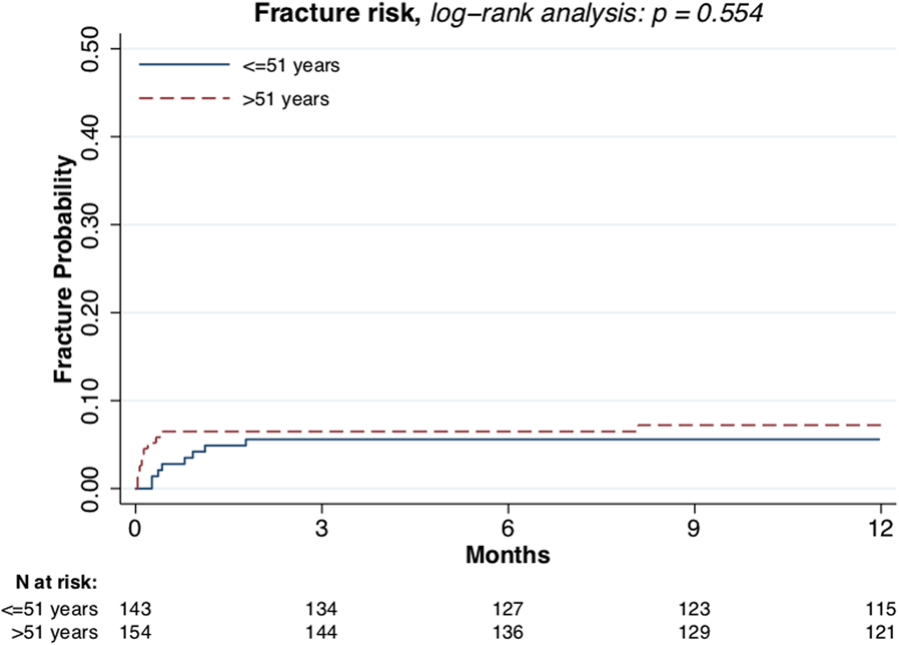
One-year fracture risk, age

**Fig. 7:**
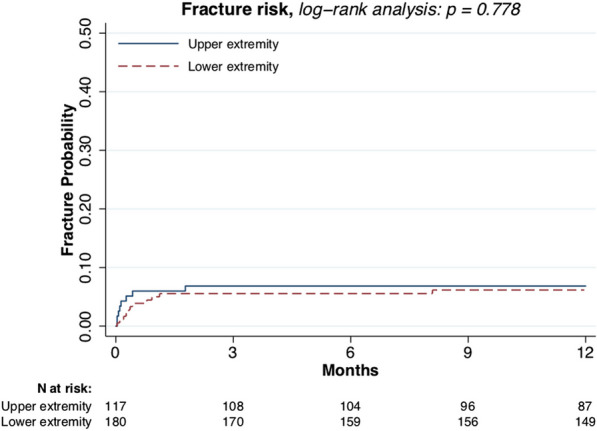
One-year fracture risk, lower and upper extremities

Male sex (*p* = 0.021, Fig. 3) and larger tumor size (*p* = 0.007, Fig. 4) were associated with higher fracture risk. Plate fixation (Fig. 5), age (Fig. 6), lower versus upper extremity (Fig. 7), and augmentation type were not associated with a higher fracture risk. Fractures following a plate fixation (*n* = 10) were all observed within the bone area covered by the plate itself.

**Fig. 8:**
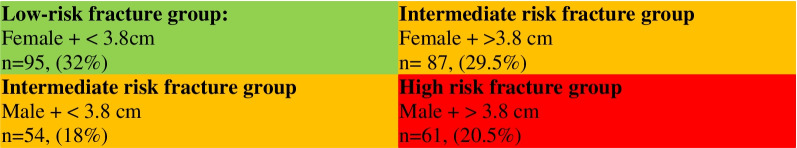
Risk groups

Stratifying the cohort based on fracture risk (i.e., sex and tumor size) demonstrates that female patients in combination with a small tumor (*n* = 95) had a 1-year fracture risk of 0.02 (95% CI 0.00–0.08) and male patients with a large tumor (*n* = 61) had a 1-year fracture risk of 0.18 (95% CI 0.10–0.30) (Fig. 3). Combining these variables low-risk, intermediate-risk, and high-risk groups are identified (Fig. 8).

## Discussion

Fractures are common after the curettage of an ACT. Intralesional curettage of an ACT weakens the bone and leads to a 6% risk of postoperative fractures. Low-, intermediate- and high-fracture risk groups were identified. Male sex and large lesions (> 3.8 cm) were strong predictors for postoperative fractures. The current knowledge of the benign behavior of ACTs in long bones, combined with the risk of complications, should discourage curettage and motivate active surveillance.

To protect patients from postoperative fractures, a large proportion of patients received postoperative weight-bearing restrictions for the first 4 to 6 weeks. The surgeon, mainly based on lesion size and location of the defect, gave these weight-bearing restrictions. Interestingly, no difference in postoperative fracture risk was found between the upper or lower extremity. This might be a result of the difference in weight-bearing restrictions given by the surgeon. In a single-blinded study of 51 patients who had been instructed to be strictly non-weight-bearing (with a mean of 24 days) after a unilateral lower-extremity surgical procedure, a non-compliance of 28% was found. Therefore, compliance should be taken into account [23]. Providing sufficient information about the weight-bearing restrictions likely improves compliance [24].

It has been observed that males exhibit a notably higher postoperative fracture risk after the curettage of an ACT. Compliance does not appear to be a factor, as differences in adherence to weight-bearing restrictions were not observed based on sex in previous studies [23]. Overall fracture risk is higher within post-menstrual females compared to males of the same age [25] while fracture risk in children is more common in boys compared to girls [26]. The patients included in this study were middle-aged and therefore represented a demographic that is somewhat in between the aforementioned groups. The fracture risk within this specific group has not been investigated previously, with plausible considerations suggesting that male-specific variables, including the possibility of higher body weight, might contribute to the susceptibility to fractures, but this remains speculative.

In line with the study of Omlor et al., no decrease in fracture risk was found by using osteosynthesis [21]. This is in contrast to a study of Deckers et al. who found a lower fracture risk for patient groups that undergo prophylactic plating compared to those who did not undergo prophylactic plating [12]. This might be caused by a confounding factor such as a larges and perhaps more fragile defect, which probably more often undergoes plate osteosynthesis. Nevertheless, the use of osteosynthesis results in 1) a more invasive first surgery and 2) a higher likelihood of secondary surgery to remove the material [21, 22]. These surgeries also come with a higher probability of complications [21].

Our study demonstrates that augmentation using allograft bone chips or PMMA after intralesional curettage of an ACT does not influence fracture risk. No significant benefit of augmentation was observed in other studies. A study by Shemesh et al. on augmentation of low-grade chondrosarcomas found a fracture risk of 17% (1/6) after allograft and 0% (0/3) after PMMA [27]. Hirn et al. identified a fracture risk of 9.6% (14/146) in patients who did not receive augmentation after curettage of benign bone tumors with various entities [28]. Chen et al. also found no difference in complication rate (including fracture risk) comparing allograft, autograft, and PMMA in 267 patients with benign bone lesions [29]. Other previous studies also demonstrated that defects consolidate without augmentation [28, 30, 31]. If augmentation does not affect fracture risk, unnecessary costs can be avoided.

Curettage of ACT became a subject of debate over the previous years [32]. ACT are categorized as benign but local aggressive lesions, and the risk of a transformation into malignant chondrosarcoma is < 1% [5, 32]. ACT can be safely differentiated from malignant chondrosarcomas using MRI [9, 18–20]. Active surveillance appeared to be a safe option, as no transformations into high-grade chondrosarcoma were observed [33]. Active surveillance did not affect patients’ well-being concerning the quality of life and has benefits on functional outcomes as compared to curettage [10, 34].

One potential disadvantage of active surveillance is the risk of complications arising from the presence of the lesion, such as pathological fractures. In our cohort, 3% of the patients had a preoperative (pathological) fracture. This is lower compared to the 13.7% seen by Alqubaisi et al. [35]. It should be noted that (pathological) fracture risk alone is not a sufficient reason to opt for curettage, as the postoperative fracture risk is described to be twice as high in our study. In a study on active surveillance through midterm MRI follow-up, 1 out of 65 patients had a fracture 24 months after diagnosis [33] which also confirmed a lower fracture risk compared to curettage outcomes. Nevertheless, it is essential to recognize that validating this active surveillance approach might require larger and more comprehensive sample sizes, along with prolonged periods of follow-up, to definitively confirm its effectiveness and long-term safety.

Limitations of our study are that the relatively low number of fractures prohibited stratified or multivariable analysis to account for potential confounding. Secondly, a cortical window was made for intralesional curettage; the size of the window likely plays an important role in fracture risk. However, the window size was not available and therefore lesion length was used as a proxy. As the sizes of the lesion and the cortical window are probably strongly related, this factor is most likely accounted for. Third, the small proportion of patients without augmentation (*n* = 27) might cause insufficient power to detect a significant difference in fracture risk between these groups. Further, the predefined follow-up of 90 days could be a potential limitation in assessing fracture risk. However, almost all patients experienced their fracture within two months and the minimum of 90-day follow-up was therefore deemed sufficient. In addition, 86% of our cohort had a minimum follow-up of 1 year.

Our study included a large patient group (297 patients) with a prolonged follow-up period. This study can function as a base for further research toward active surveillance, given the high postoperative fracture risk, especially for men with large tumor lesions.

## Conclusion

With the current knowledge of the benign behavior of ACTs in long bones and the improved radiologic modalities, postoperative fracture risk should discourage curettage and motivate active surveillance, specifically in patients with increased fracture risk (males, large-sized lesions).

### Supplementary Information

**Additional file 1: Table S1.** Baseline characteristics of patients who underwent curettage of ACT.

**Additional file 2 Table S2.** Treatment characteristics of patients who underwent curettage of ACT

**Additional file 3 Table S3.** Characteristics of patients with a fracture

## Abbreviations

ACT: Atypical cartilage tumor
IQR: Interquartile range
MRI: Magnetic resonance imaging
PMMA: Polymethyl methacrylate
WHO: World Health Organization
