# Supplementary material for: Fracture risk after intralesional curettage of atypical cartilaginous tumors
Source: J Orthop Surg Res. 2023 Nov 9;18:851. doi: 10.1186/s13018-023-04215-4 (PMC10634173; doi:10.1186/s13018-023-04215-4)
Supplement: Supplementary file 1 — Additional file 1: Table S1. Baseline characteristics of patients who underwent curettage of ACT. [file 13018_2023_4215_MOESM1_ESM.docx]

| **Table 1: Baseline characteristics of patients who underwent curettage of ACT (n=297)** | |
| --- | --- |
|  | **Mean  (±Standard Deviation)** |
| **Age** | 51 (12) |
| **Lesion size (in cm)** | 4.5 (2.8) |
|  |  |
|  | **n (%)** |
| **Male** | 114 (38) |
| **Lesion location** |  |
| Femur | 133 (45) |
| Proximal | 10 (3.4) |
| Shaft | 5 (1.7) |
| Distal | 118 (40) |
| Humerus | 94 (32) |
| Proximal | 91 (31) |
| Shaft | 3 (1.0) |
| Distal | 0 (0) |
| Tibia | 25 (8.4) |
| Proximal | 18 (6.1) |
| Shaft | 2 (0.67) |
| Distal | 5 (1.7) |
| Fibula | 20 (6.7) |
| Proximal | 19 (6.4) |
| Shaft | 0 (0) |
| Distal | 1 (0.34) |
| Hand | 20 (6.7) |
| Forearm | 3 (1.0) |
| Foot | 2 (0.67) |
| **Preoperative fracture** | 9 (3.0) |
| *ACT = Atypical Cartilage Tumor* | |
