# Supplementary material for: Fracture risk after intralesional curettage of atypical cartilaginous tumors
Source: J Orthop Surg Res. 2023 Nov 9;18:851. doi: 10.1186/s13018-023-04215-4 (PMC10634173; doi:10.1186/s13018-023-04215-4)
Supplement: Supplementary file 2 — Additional file 2 Table S2. Treatment characteristics of patients who underwent curettage of ACT [file 13018_2023_4215_MOESM2_ESM.docx]

| **Table 2: Treatment characteristics of patients who underwent curettage of ACT (n=297)** | |
| --- | --- |
|  | **n (%)** |
| **Graft** |  |
| No graft | 27 (9.1) |
| Allograft | 259 (87) |
| PMMA cement | 11 (3.7) |
| **Plate** | 110 (37) |
| **Weight bearing postoperatively** |  |
| 100% | 3 (1.0) |
| 50% | 76 (26) |
| 10% | 44 (15) |
| 0% | 108 (36) |
| Functional | 53 (18) |
| Unclear | 13 (4.4) |
| *ACT = Atypical Cartilage Tumor , PMMA = Polymethylmethacrylate.* | |
