# Supplementary material for: Fracture risk after intralesional curettage of atypical cartilaginous tumors
Source: J Orthop Surg Res. 2023 Nov 9;18:851. doi: 10.1186/s13018-023-04215-4 (PMC10634173; doi:10.1186/s13018-023-04215-4)
Supplement: Supplementary file 3 — Additional file 3 Table S3. Characteristics of patients with a fracture [file 13018_2023_4215_MOESM3_ESM.docx]

| **Table 4: Characteristics of patients with a fracture (n= 19/297)** | | | | | | | |
| --- | --- | --- | --- | --- | --- | --- | --- |
| **Case** | **Sex, Age** | **Tumor size**  **(cm)** | **Location** | **Plate** | **Augmentation** | **Weight bearing advise** | **Days until fracture (post-OR)** |
| **1** | M, 55 | 5 | Distal femur | Yes | Allograft | 50% | 1 |
| **2** | M, 46 | 7 | Distal femur | Yes | Allograft | 50% | 28 |
| **3** | M, 58 | 10 | Humerus shaft | Yes | Allograft | 50% | 4 |
| **4** | F, 56 | 4 | Proximal humerus | No | Allograft | 0% | 3 |
| **5** | F, 52 | 2 | Distal femur | No | Allograft | Functional | 13 |
| **6** | F, 71 | 5 | Proximal tibia | No | PMMA | Functional | 246 |
| **7** | M,55 | 9 | Proximal humerus | No | Allograft | 0% | 2 |
| **8** | M, 65 | 11 | Distal femur | Yes | Allograft | 50% | 6 |
| **9** | M,67 | 17 | Distal femur | No | PMMA | 50% | 3 |
| **10** | M,48 | 12 | Proximal humerus | Yes | Allograft | 0% | 8 |
| **11** | M, 49 | 3 | Proximal humerus | No | Allograft | Functional | 54 |
| **12** | M,70 | 7 | Proximal humerus | Yes | Allograft | 0% | 1 |
| **13** | F, 62 | 1 | Distal femur | No | Allograft | Tip-toe | 10 |
| **14** | F, 58 | 5 | Proximal humerus | No | Allograft | 0% | 1 |
| **15** | M, 36 | 15 | Femur shaft | Yes | Allograft | Unclear | 11 |
| **16** | M, 34 | 11 | Distal femur | Yes | Allograft | Tip-toe | 8 |
| **17** | M, 43 | 6 | Distal femur | Yes | Allograft | Tip-toe | 34 |
| **18** | F, 30 | 7 | Distal femur | Yes | Allograft | 0% | 24 |
| **19** | F, 48 | 4 | Proximal humerus | Nee | Allograft | 0% | 13 |
| *PMMA= polymethylmethacrylate, OR= Operation, F= Female, M=Male* | | | | | | | |
